# Pentobarbital Toxicity after Self-Administration of Euthasol Veterinary Euthanasia Medication

**DOI:** 10.1155/2016/6270491

**Published:** 2016-01-03

**Authors:** Steven Jason Crellin, Kenneth D. Katz

**Affiliations:** Department of Emergency Medicine, Lehigh Valley Hospital and Health Network, USF MCOM, Cedar Crest Boulevard and I-78, Allentown, PA 18103, USA

## Abstract

Suicide attempt via sodium pentobarbital is uncommon. A 48-year-old woman with a history of depression and prior suicide attempt was found unresponsive by her veterinarian spouse near a syringe containing pink solution. Upon EMS' arrival, the patient was experiencing apnea, hypoxemia, and miotic pupils; her blood glucose level measured 73 mg/dL. She was bradycardic and administered atropine with transient improvement in heart rate and transported to an emergency department; 2 mg of intravenous naloxone was administered without effect. She was endotracheally intubated via rapid sequence intubation. Rapid urine drug screening detected both benzodiazepines and barbiturates. The patient was transferred to an intensive care unit where she demonstrated a nearly absent radial pulse. Emergent fasciotomy to the left forearm and carpal tunnel was performed for acute compartment syndrome; “Euthasol” had been self-administered into the antecubital fossa. Expanded toxicological analysis via liquid chromatography/mass spectroscopy detected caffeine, atropine, 7-aminoclonazepam, phenytoin, citalopram, and naproxen. The patient's coma resolved over 48 hours and she was successfully extubated without complication. Emergency physicians must closely monitor patients exposed to veterinary euthanasia agents who develop central nervous system and respiratory depression, hypothermia, bradycardia, hypotension, or skin injury. Consultation with a regional poison center and medical toxicologist is recommended.

## 1. Introduction

Suicide attempt via self-administration of sodium pentobarbital is extremely uncommon but may occur in those who have access to veterinary medications. The dramatic presentations of barbiturate-associated poisoning are often accompanied by characteristic history and physical findings that can facilitate early identification and treatment of these potentially lethal overdoses. A unique case of a patient suffering from sodium pentobarbital toxicity after intravenous injection is described.

## 2. Case Presentation

A 48-year-old woman with a history of depression and prior suicide attempt who was prescribed clonazepam and citalopram was found by her veterinarian spouse unresponsive at home at approximately 10:30 pm. Cardiopulmonary resuscitation was initiated by the husband due to observed apnea while awaiting EMS' arrival. A syringe containing a pink solution with a needle attached was found adjacent to the patient and appeared to have been used to inject the patient's left antecubital fossa. A suicide note and an empty bottle of 2 mg clonazepam tablets were also found at the scene. Upon EMS' arrival, the patient had a Glasgow Coma Scale (GCS) score of “3” with apnea, hypoxemia, and miotic pupils. Her blood glucose level measured 73 mg/dL. She was bradycardic and was administered atropine on scene with transient improvement in heart rate.

The patient was rapidly transported to a local emergency department. Her vital signs on arrival included a heart rate of 46 beats per minute, blood pressure 99/53 mmHg, agonal respirations, temperature 94.6°F, and room air pulse oximetry of 78%. Physical examination revealed a GCS of “3,” pupils measuring 3 mm and being sluggishly reactive, and a taught, “dusky” left forearm with delayed capillary refill distal to a left antecubital vein injection site. Two milligrams of intravenous naloxone was administered without effect, and she was then endotracheally intubated via rapid sequence intubation. Computed tomography of her head demonstrated no hemorrhage, mass, or ischemic change. Serum creatinine phosphokinase and ethanol measured 806 U/L and 58 mg/dL, respectively. Complete metabolic panel, serum salicylate, and acetaminophen levels were unremarkable. Rapid urine drug screening detected both benzodiazepines and barbiturates. The patient was then transferred to a local tertiary care facility for further management.

**Figure 1:**
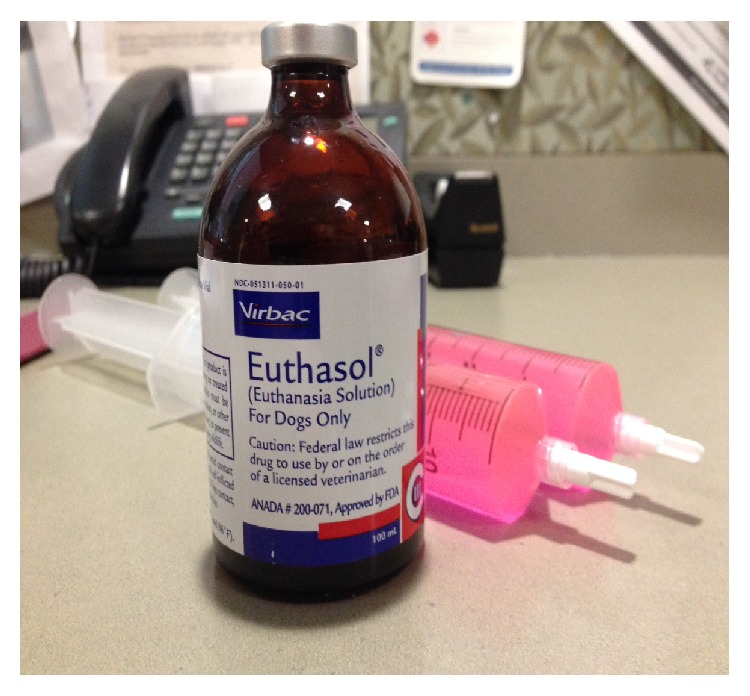
Euthasol bottle and syringes.

Upon arrival to the intensive care unit (ICU), the patient was evaluated by the surgical service. After clinical examination with a nearly absent radial pulse on Doppler examination, she underwent emergent fasciotomy of the left forearm and carpal tunnel for acute compartment syndrome. Contemporaneous discussion with her spouse revealed that the pink solution found in the syringe, which had been transported with the patient, was Euthasol (390 mg/mL pentobarbital and 50 mg/mL phenytoin; see Figure 1) and had been stolen from his on-site, secured pharmacy. The previously unused 100 mg vial was missing approximately 8 mL of solution.

Serum pentobarbital and phenytoin levels were drawn on arrival at the ICU and measured 12.6 *μ*g/mL (1.0–5.0 *μ*g/mL) and 2.5 *μ*g/mL (10–20 *μ*g/mL), respectively. Serum cell counts with differential, electrolytes, renal function, and liver enzymes were all within normal limits. Serum creatinine phosphokinase measured 1057 U/L (26–192 U/L). Expanded toxicological screening at the receiving hospital via liquid chromatography/mass spectroscopy detected caffeine, atropine, 7-aminoclonazepam, phenytoin, citalopram, and naproxen.

The patient's coma resolved over the following 48 hours, and she was successfully extubated on hospital day three. Serum pentobarbital levels declined to 8.6 (at 12 hours), 6.4 (at 24 hours), and 2.1 *μ*g/mL (at 48 hours) at time of extubation. Serum creatinine phosphokinase peaked at 4727 U/L. The plastic surgery service was consulted to repair the fasciotomy surgical wounds. The patient was ultimately evaluated by a psychiatrist, who made the diagnoses of mood disorder not otherwise specified, suicide attempt secondary to mood disorder, and alcohol use disorder. She was discharged to an inpatient facility in good condition on hospital day thirteen without neurologic sequelae.

## 3. Discussion

Pentobarbital is a short-acting barbiturate currently utilized to initiate and maintain medically induced coma, as well as for management of elevated intracranial pressure, particularly in the setting of traumatic brain injury [1]. In the United States' judicial system, the drug is utilized as a means of lethal injection in humans. Veterinarians administer pentobarbital most frequently for euthanasia purposes.

Barbiturates are derivatives of barbituric acid and are classified as either short- or long-acting. The short-acting barbiturates (e.g., pentobarbital) when compared to long-acting agents differ by more rapid onset, higher pKa values, are more protein bound and lipid soluble, have a shorter duration of action, and undergo more hepatic metabolism. Pentobarbital possesses a duration of action of three to four hours and a variable elimination half-life of 15 to 48 hours [2]. These generalizations, however, may be misleading in overdose as the severity and time course following barbiturate overdose vary greatly, and toxicokinetics are encountered rather than expected pharmacokinetics [1].

There are several distinguishing physical examination and historical features regarding barbiturate toxicity. Cutaneous manifestations of barbiturate toxicity are not uncommon and include barbiturate blisters or bullae and dermal discoloration with necrosis [3–5]. These can result from extravascular injection or prolonged external compression, both of which may be accompanied by rhabdomyolysis [6, 7]. Other well-described clinical features of barbiturate overdose are hypothermia and cardiovascular collapse, with bradycardia and refractory hypotension [8]. Furthermore, respiratory depression or embarrassment may be evident in the barbiturate-poisoned patient unlike other sedative-hypnotic medications, such as benzodiazepines. Historical clues that suggest barbiturate toxicity include access to professional veterinary euthanasia agents, as illustrated in this case [9–11]. Delayed complications of barbiturate toxicity in survivors include aspiration with and without pneumonia, pulmonary edema, cerebral edema and infarct, and multiorgan failure [1].

Concentrated solutions of sodium pentobarbital are routinely used for the purposes of animal euthanasia because of rapid onset of action and efficacy (see Table 1) [12]. Solutions often contain greater than 300 mg/mL sodium pentobarbital. Intravenous injection in humans and animals results in rapid coma, respiratory depression, hypotension, bradycardia, and hypothermia. These effects lead to prompt induction of asystole and death. While the majority of these agents are administered intravenously, they are also efficacious via oral, intramuscular, or intraperitoneal routes. It has been reported that oral doses of sodium pentobarbital as little as 100 mg can induce hypnosis in adults, which substantially increases the lethality of unsecured concentrated veterinary formulations [10].

Concentrated veterinary solutions (and lower-dose formulations utilized for human use) often contain chemical vehicles, which—while facilitating administration of the medication—can also have significant side effects. These diluents are often alcohol derivatives, for instance, isopropanol, ethanol, and propylene glycol. Side effects of these additives include potentiation of the sedative-hypnotic effects of the barbiturate and metabolic derangements such as lactic acidosis documented from propylene glycol additives [13].

Several formulations of pentobarbital-containing veterinary euthanasia agents have varying types of Vaughan-Williams Class-Ib antiarrhythmics, most frequently phenytoin sodium. According to the Euthasol package insert, the rapid administration of these agents reportedly produces cardiotoxic effects during the anesthesia stage of euthanasia by “hastening the cessation of electrical activity of the heart” via interference with myocardial sodium channel function. In this patient, the clinical toxicity observed stemmed directly from sedative-hypnotic toxicity and likely very little from the phenytoin.

Similar to other medications in the sedative-hypnotic class, the treatment for barbiturate overdose remains primarily supportive. If toxic amounts were ingested orally, activated charcoal could be considered, but, given the potential for significant CNS depression and subsequent aspiration, the benefit appears small [14]. Pentobarbital metabolism occurs primarily via hydroxylation and glucuronidation in the liver, and excretion occurs primarily in the kidneys. Alkaline diuresis does not enhance excretion because of the high pKa of the short-acting barbiturates [10]. Hemodialysis appears to be less effective for the short-acting agents compared to long-acting ones and is described as “moderately dialyzable” [2, 10, 15]. The patient described in this case report improved spontaneously with meticulous supportive care and maintained normal renal function, so hemodialysis was deemed unnecessary.

Pentobarbital poisoning is extremely uncommon but still can be lethal. Emergency physicians must be cognizant of patients who develop CNS and respiratory depression, hypothermia, bradycardia, hypotension, or skin injury, especially in the setting of exposure to veterinary euthanasia agents. Meticulous intensive care is the mainstay of treatment with attention to respiratory and cardiovascular support, and high flux hemodialysis may enhance elimination in severe, refractory cases. Consultation with both a regional poison center and a medical toxicologist is recommended.

## Figures and Tables

**Table 1:** Pentobarbital-containing veterinary euthanasia solutions [12].

Pentobarbital-containing veterinary euthanasia solutions			
	Concentrations	Trade names	Veterinary uses
Pentobarbital sodium powder	392 mg/mL when reconstituted in 250 mL	*Fatal-Plus Powder* (Vortech) *Pentasol Powder* (Virbac)	Approved for all warm-blooded animals

Pentobarbital sodium for injection	260 mg/mL in 100 mL bottles	*Sleepaway* (Fort Dodge)	Approved for use in dogs and cats
	389 mg/mL in 100 mL or 250 mL vials	*Socumb-6 gr* (Butler) *Somlethal* (Webster)	Approved for use in dogs and cats
	390 mg/mL in 250 mL vials	*Fatal-Plus* Solution (Vortech)	Approved for use in dogs and cats

Pentobarbital sodium/phenytoin sodium	390 mg/mL pentobarbital sodium and 50 mg/mL phenytoin sodium in 100 mL vials	*Beuthanasia*-*D Special* (Schering-Plough) *Euthasol* (Virbac)	Approved for use in dogs
